# Muscle Androgen Receptor Content but Not Systemic Hormones Is Associated With Resistance Training-Induced Skeletal Muscle Hypertrophy in Healthy, Young Men

**DOI:** 10.3389/fphys.2018.01373

**Published:** 2018-10-09

**Authors:** Robert W. Morton, Koji Sato, Michael P. B. Gallaugher, Sara Y. Oikawa, Paul D. McNicholas, Satoshi Fujita, Stuart M. Phillips

**Affiliations:** ^1^Department of Kinesiology, McMaster University, Hamilton, ON, Canada; ^2^Graduate School of Human Development and Environment, Kobe University, Kobe, Japan; ^3^Department of Mathematics and Statistics, McMaster University, Hamilton, ON, Canada; ^4^College of Sport and Health Sciences, Ritsumeikan University, Shiga, Japan

**Keywords:** resistance exercise, testosterone, intramuscular, androgen receptor, hypertrophy

## Abstract

The factors that underpin heterogeneity in muscle hypertrophy following resistance exercise training (RET) remain largely unknown. We examined circulating hormones, intramuscular hormones, and intramuscular hormone-related variables in resistance-trained men before and after 12 weeks of RET. Backward elimination and principal component regression evaluated the statistical significance of proposed circulating anabolic hormones (e.g., testosterone, free testosterone, dehydroepiandrosterone, dihydrotestosterone, insulin-like growth factor-1, free insulin-like growth factor-1, luteinizing hormone, and growth hormone) and RET-induced changes in muscle mass (*n* = 49). Immunoblots and immunoassays were used to evaluate intramuscular free testosterone levels, dihydrotestosterone levels, 5α-reductase expression, and androgen receptor content in the highest- (HIR; *n* = 10) and lowest- (LOR; *n* = 10) responders to the 12 weeks of RET. No hormone measured before exercise, after exercise, pre-intervention, or post-intervention was consistently significant or consistently selected in the final model for the change in: type 1 cross sectional area (CSA), type 2 CSA, or fat- and bone-free mass (LBM). Principal component analysis did not result in large dimension reduction and principal component regression was no more effective than unadjusted regression analyses. No hormone measured in the blood or muscle was different between HIR and LOR. The steroidogenic enzyme 5α-reductase increased following RET in the HIR (*P* < 0.01) but not the LOR (*P* = 0.32). Androgen receptor content was unchanged with RET but was higher at all times in HIR. Unlike intramuscular free testosterone, dihydrotestosterone, or 5α-reductase, there was a linear relationship between androgen receptor content and change in LBM (*P* < 0.01), type 1 CSA (*P* < 0.05), and type 2 CSA (*P* < 0.01) both pre- and post-intervention. These results indicate that intramuscular androgen receptor content, but neither circulating nor intramuscular hormones (or the enzymes regulating their intramuscular production), influence skeletal muscle hypertrophy following RET in previously trained young men.

## Introduction

There is substantial individual variability in RET-induced skeletal muscle hypertrophy (Hubal et al., 2005; Davidsen et al., 2011). The post-exercise rise in circulating, presumably anabolic, hormones (e.g., T, GH, and IGF-1) are believed to be causative in determining RET-induced skeletal muscle hypertrophy (Kraemer et al., 2017; Mangine et al., 2017). However, there is substantial contrary evidence for a causal, or even related (i.e., sharing common variance) role of such hormones in both RET-induced increases in muscle protein synthesis (West et al., 2009) and hypertrophy (West et al., 2010; West and Phillips, 2012; Mitchell et al., 2013; Morton et al., 2016; Mobley et al., 2018).

It is plausible that, as opposed to systemic circulating hormones, local intramuscular androgenesis could mediate RET-induced muscle hypertrophy as has been proposed for older men (Sato et al., 2014). In addition, the RET-induced increase in intramuscular androgen receptor content has been significantly correlated with RET-induced muscle hypertrophy (Ahtiainen et al., 2011; Mitchell et al., 2013). Thus, it may be that an increase in intramuscular androgens and/or their receptors, via an autocrine mechanism, are important in determining RET-induced hypertrophy.

The purpose of this study was to determine if the heterogeneity in RET-induced skeletal muscle hypertrophy, measured using multiple indices, was associated with circulating hormones, intramuscular hormones, intramuscular steroidogenic enzyme content, or androgen receptor content. We performed additional statistical and intramuscular analyses on data from a previous study in healthy, resistance-trained men (*n* = 49; Morton et al., 2016). To further explore the relationship between systemic hormones and hypertrophy we used backward elimination and principal component regression on systemic hormone concentrations both at rest and post-resistance exercise with indices of hypertrophy as separate outcome variables in all participants. To evaluate the significance of intramuscular androgenesis we completed an analysis on only our highest- (HIR – top quintile) and lowest- (LOR – bottom quintile) responders that included evaluation of intramuscular T, DHT, 5α-reductase expression, and androgen receptor content. Consistent with our previous work (West et al., 2010; West and Phillips, 2012; Mitchell et al., 2013; Morton et al., 2016), we hypothesized that circulating systemic hormones would not be related to any measure of hypertrophy; however, we hypothesized, given previous findings (Ahtiainen et al., 2011; Mitchell et al., 2013), that androgen receptor content would be associated with RET-induced hypertrophy.

## Materials and Methods

### Participants and Resistance Exercise Training Intervention

Forty-nine resistance-trained (performing RET at least 2 days/week [range 3–6 days/week] for 4 ± 6 years) young men volunteered for this study. Each participant was informed of associated risks with the RET intervention and testing and the study was carried out in accordance with the recommendations of the most recent Tri-Council statement on research in human participants^1^. The protocol was approved by the Hamilton Integrated Research Ethics Board and all subjects gave written informed consent in accordance with the Declaration of Helsinki. The trial was registered at https://clinicaltrials.gov/ as NCT02139865. An overview of the RET intervention can be read in detail in the original manuscript (Morton et al., 2016). Briefly, participants were randomly allocated to either a high repetition (HR) or low repetition (LR) group. The HR group performed all exercises with relatively light resistance [∼30–50% of their repetition maximum (RM)] until volitional failure (20–25 repetitions) and the LR group performed all exercises with relatively heavy resistance (∼75–90% RM), also until volitional failure (8–12 repetitions). Each participant underwent a 12-week RET intervention where they performed whole-body RET 4 days/week and received 30 g of whey protein isolate twice per day (BioPRO; Davisco Foods International, Le Sueur, MN, United States).

### Blood Collection and Hormone Analysis

The pre- and post-intervention testing day was performed after an overnight fast at the same time of day for each participant. Each participant performed an acute bout of resistance exercise within their designated group assignment (HR or LR) and blood was drawn from an intravenous catheter inserted in an antecubital vein. Two 4 mL vacutainer tubes (Becton, Dickinson and Company, Franklin Lakes, NJ, United States) were collected pre-exercise and 0-, 15-, 30-, and 60-min post-exercise. One 4 mL tube was allowed to clot for 30 min at room temperature to later isolate serum and the other was heparinized to later isolate plasma. Blood sample analysis was done blinded for: cortisol (nM), LH (IU/L), lactate (mM), DHEA (ng/mL), T (ng/mL), free T (fT; ng/dL; i.e., testosterone that is not bound to sex hormone-binding globulin or albumin in the blood), DHT (ng/mL), and GH (ng/mL) using solid-phase, two site chemiluminescence immunometric assays (Immulite 2000 Immunoassay System; Siemens Healthineers, Erlangen, Germany) and IGF-1 (μg/dL) and free IGF-1 (fIGF-1; ng/mL) using radio-immunoassays (Diagnostics Products Corporation, Los Angeles, CA, United States). The 60-min post-resistance exercise AUC was calculated for each hormone, using the trapezoidal rule, with time points at 0, 15, 30, and 60 min.

### Stepwise Regressions

HR and LR data were collapsed due to a lack of difference in both circulating hormones and outcomes between-groups (Morton et al., 2016). The outcomes considered were type 1 fiber CSA, type 2 fiber CSA, and fat- and bone-free (lean) body mass (LBM). Each outcome at each time of measurement (i.e., the change, absolute pre-, and absolute post-intervention values) were regressed against hormones from each time point: pre-intervention resting, pre-intervention post-exercise AUC, post-intervention resting, and post-intervention post-exercise AUC. Backward elimination, with the Akaike Information Criterion (AIC) as the elimination criterion, was used to choose the final model. The post-exercise AUC values used in the analysis did not subtract out the resting concentrations. We did, however, run the analysis with the resting concentrations subtracted from the AUC raw values and there were no major differences in our results.

### Immunoblot Analysis

As previously described (Aizawa et al., 2010), after homogenization, the protein concentration of resulting supernatant was determined by a Bradford protein assay, and muscle proteins (both cytoplasmic and nuclear, 20 μg protein) were separated on 10% SDS-polyacrylamide gels and then transferred to polyvinylidene difluoride membranes (Millipore, Billerica, MA, United States). The membranes were blocked for 1 h with blocking buffer (5% skim milk in phosphate-buffered saline with 0.1% Tween 20) and then incubated for 12 h at 4°C with primary antibodies against androgen receptor (#3202, Cell Signaling Technology, Beverly, MA, United States) and 5α-reductase (H00006715, Abnova, Taipei, Taiwan) diluted to 1:1000 in blocking buffer. The membranes were washed three times with PBST before being incubated for 1 h with a horseradish peroxidase-conjugated secondary antibody and anti-rabbit immunoglobulin (#7074, Cell Signaling Technology, Beverly, MA, United States) diluted to 1:3000 in the blocking buffer. The membranes were then washed with PBST three times. The proteins were detected using an enhanced chemiluminescence plus system (GE Healthcare Biosciences) and visualized on an LAS4000 imager (GE Healthcare Biosciences). Band intensities were quantified using ImageJ version 1.46 (National Institutes of Health, Bethesda, MD, United States).

### Enzyme Immunoassays for Intramuscular Hormones

Muscle sample was homogenized using the same method as the immunoblot analysis. The levels of T and DHT in skeletal muscle were determined using an enzyme-linked immunosorbent assay kit, after being diluted 200 times with each assay buffer as previously described (Horii et al., 2016). The immobilized polyclonal antibodies were raised against T (Cayman Chemical, Ann Arbor, MI, United States) and DHT (IBL Hamburg, Germany) before secondary horseradish peroxidase antibodies were added. Optical density at 450 nm was qualified on a microplate reader (BioLumin 960; Molecular Dynamics, Tokyo, Japan) and were assayed in duplicate. The coefficient of variation value was 3.0 and *r*^2^ = 0.974 in the present study. The researchers that performed the intramuscular analyses (KS and SF) were not blinded to which samples were HIR and LOR.

### Principal Component Analysis and Regression

The data were centered and scaled before principal component analysis (PCA) was performed on the hormones from each time of measurement (pre-intervention resting, pre-intervention post-exercise AUC, post-intervention resting, and post-intervention post-exercise AUC). The purpose of PCA is to use orthogonal transformation to create a set of new linear, uncorrelated variables (principal components), a subset of which is taken that effectively accounts for most of the variability seen in the original data. Ultimately, these principal components are linear combinations of the original variables (e.g., hormones) that are later used as covariates in regression analyses herein. We present the PCA here in scree plots. Backward elimination was performed on the principal components (i.e., principal component regression) using AIC as the model fit criterion. PCA and principal component regression were performed in R (R Core Team, 2017).

### High- vs. Low-Responders

**FIGURE 1:**
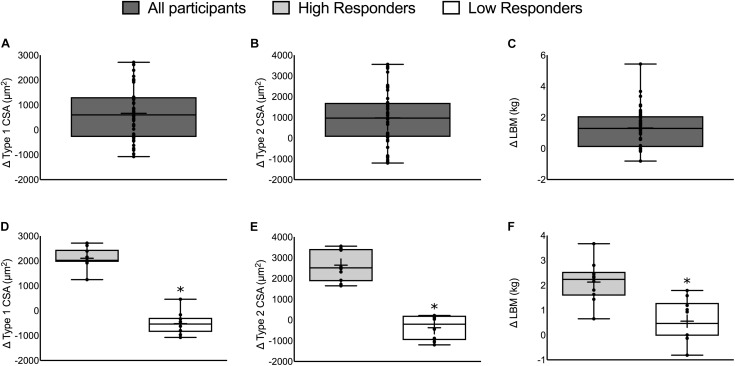
The change in muscle mass in all participants (top) and HIR and LOR (bottom). Top panels: The change in **(A)** type 1 CSA, **(B)** type 2 CSA, and **(C)** LBM from all 49 participants. Bottom panels: The change in **(D)** type 1 CSA, **(E)** type 2 CSA, and **(F)** LBM categorized into HIR and LOR. Values are presented as median (lines) with interquartile range (boxes), range (minimum and maximum), and mean (cross). ^∗^Significant difference between high- and low-responders (*P* < 0.01). Panels **A–C** adapted from Morton et al. (2016).

Skeletal muscle biopsies from each participant’s *vastus lateralis* and DXA were used to assess the change in fiber CSA (both type 1 and type 2) and LBM, respectively, as described in detail elsewhere (Morton et al., 2016). The determination of HIR and LOR was done by individually ranking (from 1 to 49) the change in each outcome for each participant and then averaging each participant’s rank across all three outcomes (type 1 CSA, type 2 CSA, and LBM). With a probability of type II error (*alpha*) of 0.05, a type I error probability (*beta*) of 0.20, and a relatively moderate expected difference in RET-induced changes in muscle mass between HIR and LOR (effect size, *f* = 0.60), *a priori* sample size calculations required 18 participants (nine in each group). Thus, the top quintile (*n* = 10) of ranked participants were categorized as the HIR and the bottom quintile (*n* = 10) of ranked participants were categorized as the LOR. Statistical analyses between HIR and LOR was performed using SPSS (version 22.0, Chicago, IL, United States). Type 1 CSA, type 2 CSA, LBM, and all intramuscular hormone-related data were analyzed using a two-factor (group × time) repeated measures analysis of variance (ANOVA) with group (HIR vs. LOR) and time (pre- vs. post-intervention) as the experimental variables. If indicated, independent two-tailed *t*-tests were run to evaluate any differences between-groups at a specific time point (e.g., pre-intervention intramuscular T). Correlations between intramuscular outcomes and the change in type 1 CSA, type 2 CSA, and LBM were performed in SPSS (version 22.0, Chicago, IL, United States). Statistical significance was accepted when *P* < 0.05. Data are presented as box and whisker plots (including the median [line], mean [cross], interquartile range [box], and minimum and maximum values [whiskers]) in **Figures 1, 3** and mean ± SD in text and tables.

## Results

### Changes in Muscle Mass With Resistance Exercise Training

Fifty-six participants were recruited and 49 participants completed the whole intervention (HR: *n* = 24, LR: *n* = 25; 23 ± 2 years, 86 ± 5 kg, 181 ± 6 cm). Two individuals dropped out from the LR group due to work relocation and a non-intervention related injury and five individuals dropped out of the HR group due to either a change in location or a non-intervention related injury. Twelve weeks of RET resulted in an increase in type 1 CSA (665 ± 149 μm^2^), type 2 CSA (978 ± 189 μm^2^), and LBM (1.22 ± 1.37 kg, *P* < 0.01; **Figures 1A–C**, respectively; Morton et al., 2016). There were no differences between repetition groups (HR versus LR – see Morton et al., 2016) for any of the outcomes.

### Stepwise Regressions

For each outcome (change in type 1 CSA, type 2 CSA, and LBM) none of the post-exercise AUC (**Table 1**) or the resting concentrations (**Table 2**) of any hormone measured either pre- or post-intervention were consistently significant (i.e., significant with multiple outcomes or at more than one time of measurement) in the final models. Furthermore, the coefficients of determination (i.e., *R*^2^) values were low (<0.25) for all outcomes at each time of measurement indicating that little of the variation seen in the hypertrophic response can be explained by any model fitted here. Similar results were found when evaluating the pre- and post-intervention type 1 CSA, type 2 CSA, and LBM against resting hormone concentrations (**Supplementary Table 1**).

**Table 1:** Backward elimination regression final output between post-exercise systemic hormone AUC and the change in type 1 CSA, type 2 CSA, and LBM.

	Pre-intervention post-exercise AUC					Post-intervention post-exercise AUC			
			
	Estimate	SEM	*t*-Value	*p*-Value		Estimate	SEM	*t*-Value	*p*-Value
**Δ Type 1 CSA**					**Δ Type 1 CSA**				
Intercept	636	160	4.0	0.01	Intercept	669	145	4.6	0.01
DHEA	-230	162	-1.4	0.16	DHT	-239	147	-1.6	0.11
					fIGF-1	305	147	2.1	0.04
	*F = 2.03*	*df = 42*	*R^2^= 0.05*	*pv = 0.16*		*F = 3.27*	*df = 45*	*R^2^= 0.13*	*pv = 0.05*
**Δ Type 2 CSA**					**Δ Type 2 CSA**				
Intercept	949	184	5.2	0.01	Intercept	982	190	5.2	0.01
LH	-508	197	-2.6	0.01	fT	-337	200	-1.7	0.10
GH	371	199	1.9	0.07	DHEA	-287	200	-1.4	0.16
DHEA	-287	188	-1.5	0.14					
	*F = 3.63*	*df = 40*	*R^2^= 0.21*	*pv = 0.02*		*F = 1.93*	*df = 45*	*R^2^= 0.08*	*pv = 0.16*
**Δ LBM**					**Δ LBM**				
Intercept	1.2	0.2	6.0	0.01	Intercept	1.2	0.2	6.3	0.01
fIGF-1	0.3	0.2	1.6	0.12	DHT	-0.3	0.2	-1.4	0.17
					Lactate	-0.4	0.2	-2.0	0.05
	*F = 2.54*	*df = 42*	*R^2^= 0.06*	*pv = 0.12*		*F = 2.67*	*df = 45*	*R^2^= 0.11*	*pv = 0.08*


**Table 2:** Backward elimination regression final output between resting hormones and the change in type 1 CSA, type 2 CSA, and LBM.

	Pre-intervention resting					Post-intervention resting			
			
	Estimate	SEM	*t*-Value	*p*-Value		Estimate	SEM	*t*-Value	*p*-Value
**Δ Type 1 CSA**					**Δ Type 1 CSA**				
Intercept	667	147	4.6	0.01	Intercept	667	140	4.8	0.01
IGF-1	232	148	1.6	0.12	T	-207	143	-1.4	0.16
	*F = 2.45*	*df = 47*	*R^2^= 0.03*	*pv = 0.12*	LH	-258	143	1.8	0.08
					Cortisol	-218	143	-1.5	0.13
						*F = 2.93*	*df = 45*	*R^2^= 0.16*	*pv = 0.04*
**Δ Type 2 CSA**					**Δ Type 2 CSA**				
Intercept	978	182	5.4	0.01	Intercept	978	183	5.4	0.01
LH	-403	186	-2.2	0.04	LH	-327	185	-1.8	0.08
GH	293	186	1.6	0.12	Cortisol	-283	185	-1.5	0.13
	*F = 3.10*	*df = 46*	*R^2^= 0.12*	*pv = 0.06*		*F = 2.76*	*df = 46*	*R^2^= 0.11*	*pv = 0.07*
**Δ LBM**					**Δ LBM**				
Intercept	1.2	0.2	6.8	0.01	Intercept	1.2	0.2	6.8	0.01
DHT	-0.4	0.2	-2.2	0.03	fT	0.3	0.2	1.7	0.11
Lactate	-0.3	0.2	-1.7	0.09	DHT	-0.3	0.2	-1.8	0.09
Cortisol	0.4	0.2	2.0	0.06	GH	0.4	0.2	1.9	0.06
	*F = 3.84*	*df = 45*	*R^2^= 0.20*	*pv = 0.02*		*F = 4.26*	*df = 45*	*R^2^= 0.22*	*pv = 0.01*


### Principal Component Analysis

**FIGURE 2:**
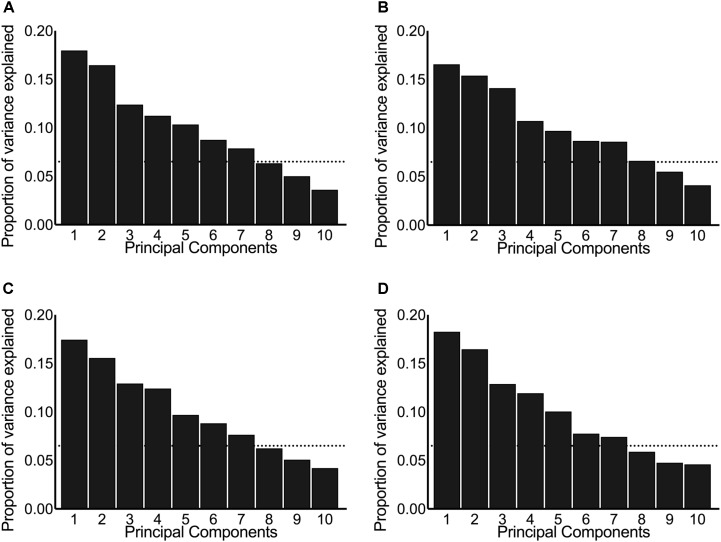
Scree plots illustrating the proportion of variance in the original hormones explained by each principal component. The panels include the principal components derived from systemic hormones measured: **(A)** pre-intervention post-exercise, **(B)** post-intervention post-exercise, **(C)** pre-intervention resting, and **(D)** post-intervention resting. The dotted horizontal line indicates the cut-off point where the principal components above explained ≥80% variance between the original data set of hormones.

Principal component analysis was performed on centered and scaled predictors and is presented here as scree plots for the pre-intervention post-exercise AUC (**Figure 2A**), post-intervention post-exercise AUC (**Figure 2B**), pre-intervention resting concentrations (**Figure 2C**), and post-intervention resting concentrations (**Figure 2D**). As illustrated by the shallow-sloped scree plots, no principal component was particularly effective at explaining variance in the original data set.

We chose to keep the number of principal components that explain ≥80% of the variance in the original predictors, which resulted in seven principal components included in each of our principal component stepwise regressions. Running principal component stepwise regression (regardless of whether the hormones were evaluated at rest, post-exercise, pre-intervention, or post-intervention) revealed that no principal component was consistently significant or consistently included in any of the final models and that the final *R*^2^ never exceeded 0.25 and was as low as 0.05 (**Supplementary Tables 2–4**). These results indicate that very little of the variation seen in the hypertrophic response to RET can be explained by any of the fitted models.

### High- vs. Low-Responders

There was a significant difference between HIR and LOR in the change in type 1 CSA (HIR: 2106 ± 412, LOR: -520 ± 450 μm^2^), type 2 CSA (HIR: 2642 ± 756, LOR: -373 ± 593 μm^2^), and LBM (HIR: 2.1 ± 0.8, LOR: 0.6 ± 0.8 kg, *P* ≤ 0.001; **Figures 1D–F**). There was no difference in the number of participants from each training group (HIR: four and six and LOR: six and four from HR and LR, respectively).

There was no difference in any resting hormone concentration between HIR and LOR with the exception of the post-intervention resting concentration of LH (HIR: 3.67 ± 0.63; LOR 4.59 ± 1.15 IU/L, *P* < 0.01) and lactate (HIR: 0.52 ± 0.05; LOR: 0.55 ± 0.07 mM, *P* = 0.02), which were greater in the LOR. There was no difference in the post-exercise AUC for any hormone between HIR and LOR with the exception of pre-intervention post-exercise cortisol, which was higher in the HIR (HIR: 576 ± 100; LOR: 508 ± 199 nM; *P* < 0.001).

### Intramuscular Hormones

**FIGURE 3:**
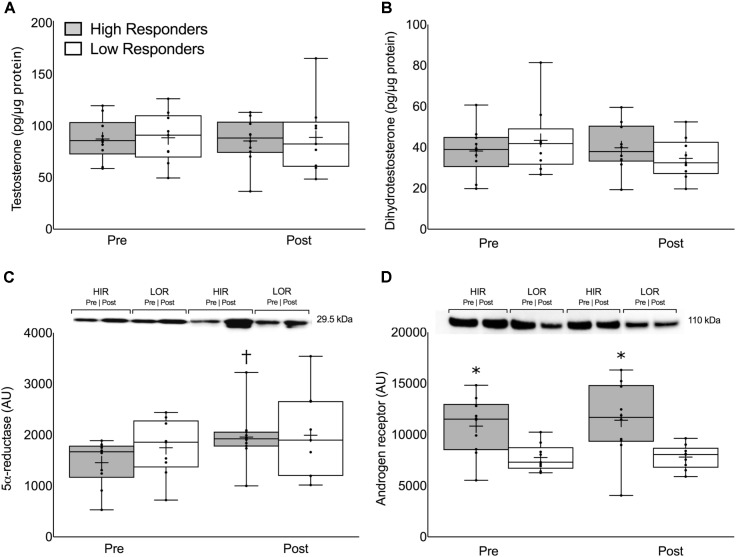
Intramuscular **(A)** free testosterone concentration, **(B)** dihydrotestosterone concentration, **(C)** 5α-reductase expression, and **(D)** androgen receptor content presented pre- and post-intervention in both high- and low-responders. Representative immunoblots for 5α-reductase expression **(C)** and androgen receptor content **(D)** are shown between HIR and LOR both pre- and post-intervention with their respective weights. Values are presented as median (lines) with interquartile range (boxes), range (minimum and maximum), and mean (cross). ^†^Significant difference between pre- and post-intervention (*P* < 0.01). ^∗^Significant difference between high- and low-responders (*P* < 0.05).

There were no differences in the pre-intervention, post-intervention, or change in intramuscular T or DHT between HIR and LOR (**Figures 3A,B**, respectively). The change in 5α-reductase expression was significant in HIR (pre: 1457 ± 450, post: 1957 ± 543 AU, *P* < 0.01) but not in LOR (pre: 1748 ± 559, post: 1994 ± 840 AU, *P* = 0.32; **Figure 3C**). The pre-intervention (HIR: 10827 ± 2789, LOR: 7759 ± 1323 AU, *P* < 0.01) and post-intervention (HIR: 11406 ± 2789, LOR: 7801 ± 1189 AU, *P* = 0.01; **Figure 3D**) intramuscular androgen receptor content was significantly greater in HIR versus LOR. There was no change in intramuscular androgen receptor content pre- to post-intervention (Δ319 ± 1314 AU, *P* = 0.75) and there was a linear relationship between the participants’ pre- and post-intervention androgen receptor content (*r* = 0.92).

**FIGURE 4:**
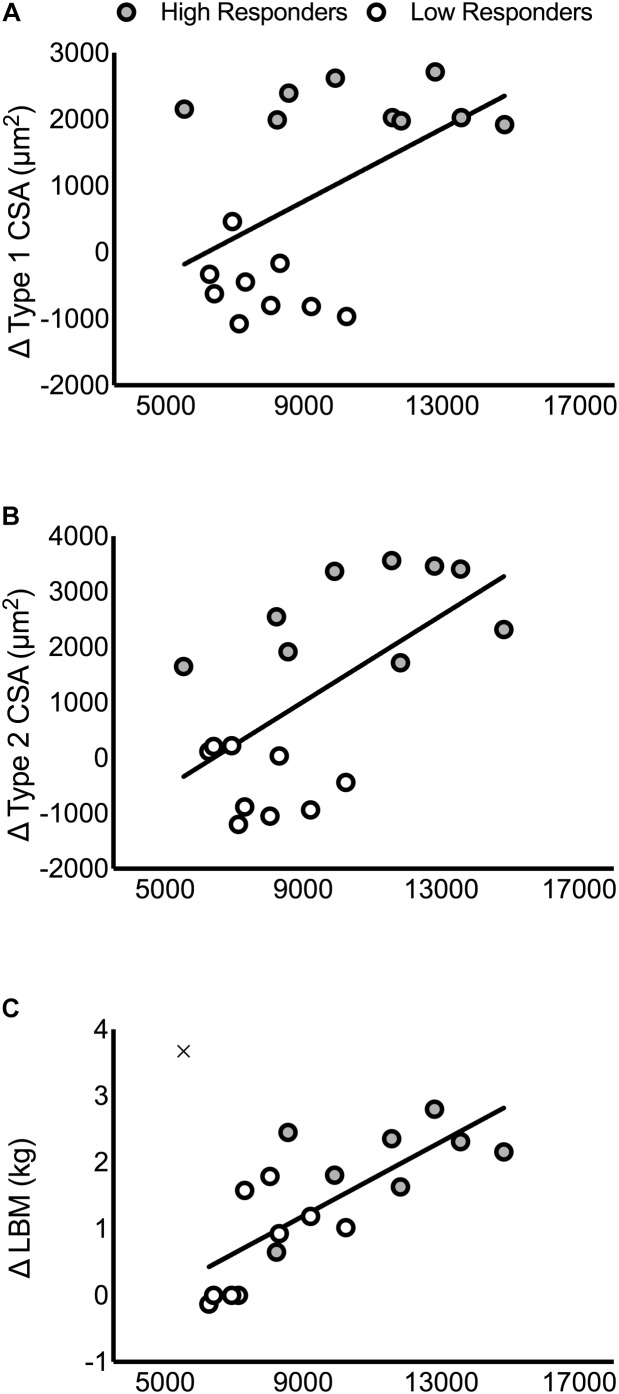
Correlations between the pre-intervention intramuscular androgen receptor content and changes in muscle mass. Correlations are presented in panels for: **(A)** type 1 CSA (*r* = 0.51, *P* = 0.03), **(B)** type 2 CSA (*r* = 0.61, *P* < 0.01), and **(C)** LBM (*r* = 0.76, *P* < 0.01). In **(C)**, the outlier that was removed from the correlational analysis between pre-intervention androgen receptor content and LBM is included on the figure as an ‘×.’

There were no significant correlations between the pre-intervention, post-intervention, or change in intramuscular T, DHT, or 5α-reductase with the change in type 1 CSA, type 2 CSA, or LBM (*P* > 0.05; **Supplementary Table 5**). In contrast, pre-intervention, post-intervention, and the average between pre- and post-intervention androgen receptor content was significantly correlated with the change in LBM (pre: *r* = 0.76, *P* < 0.01; post: *r* = 0.75, *P* < 0.01; average: *r* = 0.77, *P* < 0.01), type 1 CSA (pre: *r* = 0.51, *P* = 0.03; post: *r* = 0.49, *P* = 0.04; average: *r* = 0.51, *P* = 0.03), and type 2 CSA (pre: *r* = 0.61, *P* < 0.01; post: *r* = 0.65, *P* < 0.01; average: *r* = 0.64, *P* < 0.01; **Supplementary Table 5** and **Figure 4**). One participant’s data was removed from the regression analyses that included the change in LBM because it was identified as a statistical outlier via the robust regression and outlier removal method at a coefficient of 1% (Motulsky and Brown, 2006). We have indicated the location of this participant in **Figure 4** for illustrative purposes.

## Discussion

The main finding of the present study, consistent with our previous work, was that no systemic hormone shared significant variance with RET-induced changes in skeletal muscle fiber CSA or skeletal muscle mass in resistance-trained men (**Tables 1, 2**). We extend these findings to include local muscle-measured hormonal concentrations, which also did not show a significant association with any index of hypertrophy. We found that HIR had increased 5α-reductase content following 12 weeks of RET and had significantly higher androgen receptor content, which did not change with RET, than LOR both prior to- and after-RET (**Figure 3**). We conclude that neither systemic nor local muscular hormone availability influence RET-induced skeletal muscle hypertrophy in healthy young men. Consistent with previous work, we propose instead that the magnitude of RET-induced skeletal muscle hypertrophy is modulated in part by intramuscular androgen receptor content (**Figure 4**) and likely other intramuscular variables.

### Circulating Hormones and Resistance Exercise Training

Recent publications (Kraemer et al., 2017; Mangine et al., 2017) and guidelines (Ratamess et al., 2009) claim that circulating hormones are mechanistically and directly related to, and predictive of, RET-induced changes in skeletal muscle mass despite contrary evidence that they are not (West et al., 2010; West and Phillips, 2012; Mitchell et al., 2013; Morton et al., 2016; Mobley et al., 2018). In a previous study, we ran 120 correlations, each on 49 participants, between 10 different hormones and various measures of changes in muscle mass and strength. We found that only the post-exercise rise in cortisol was correlated with changes in type 2 CSA (pre-intervention: *r* = -0.34, *P* = 0.02; post-intervention: *r* = -0.31, *P* = 0.04) (Morton et al., 2016). Others have found significant correlations between the post-exercise rise in circulating GH (McCall et al., 1999) and T (Ahtiainen et al., 2003; Brook et al., 2016) with changes in muscle mass but those correlations were run on samples consisting of less than 11 participants, which could give rise to spurious correlations. Here, we ran an additional 48 stepwise regressions from 49 participants, 10 hormones, and three separate hypertrophy-related outcomes including muscle fiber size. We found that no hormone was consistently significant, nor did any final model have a high coefficient of determination, i.e., all *R*^2^ values were below 0.25. Moreover, PCA was not effective at reducing the total variance amongst the original hormone data (**Figure 2**) and there was no regression model with the principal components used as covariates that explained a meaningful proportion of the variability in any outcome (**Supplementary Tables 2–4**). There is now substantial evidence to suggest that circulating systemic hormones measured at rest (McCall et al., 1999; Morton et al., 2016; Mobley et al., 2018) and/or post-exercise (Ahtiainen et al., 2003; West et al., 2010; West and Phillips, 2012; Mitchell et al., 2013; Morton et al., 2016) share no common variance and are thus neither related to nor predictive of RET-induced changes in muscle mass in healthy young participants.

A recent study (Mangine et al., 2017) used partial least squares-structural equation modeling (PLS-SEM) and reported that a model with composite hormonal scores (T, GH, IGF-1, insulin, and cortisol) and a composite measure of hypertrophy (muscle CSA and thickness from the vastus lateralis and rectus femoris) resulted in a significant coefficient of determination (*R*^2^= 0.73). The interpretation of this finding was that the composite hormonal score was related to a composite score of hypertrophy. What is troubling with this interpretation is that the model without T (the model’s best hormonal predictor) still had a substantial coefficient of determination (*R*^2^ = 0.43) with the hypertrophy composite score and was statistically significant. In fact, individual removal of the other hormones (GH, IGF-1, insulin, and cortisol) showed negligible effect on the shared variance of the model and yet the model *without* its ‘best’ predictive hormone, T, accounted for almost 60% of the variance seen *with* that hormone present in the model. While the authors argued for unexplained interactions between hormones as being a reason for the model variance without T, we suggest it is more likely that PLS weights capitalize on chance to exaggerate the correlations (Goodhue et al., 2012). While we see value in PLS-SEM for examination of large datasets, there are substantial limitations to interpretation when small sample sizes (*n* = 26) are used (Goodhue et al., 2012). Defining PLS as an appropriate SEM method has also been called into question for estimation and inference (Rönkkö and Evermann, 2013) and the coefficient of determination (e.g., *R*^2^) is a poor yardstick for assessing PLS-SEM model fit because inconsistent estimators can produce models with high *R*^2^. Consequently, not all well-fit models are predictive (Henseler et al., 2014) and not all predictive models are well-fit (McIntosh et al., 2014).

### High- vs. Low-Responders to Resistance Exercise Training

To investigate potential determinants of the heterogeneity in RET-induced skeletal muscle hypertrophy (Hubal et al., 2005; Davidsen et al., 2011; Morton et al., 2016), we stratified 49 participants into HIR (*n* = 10) and LOR (*n* = 10) based on their change in three indicators of skeletal muscle mass (type 1 CSA, type 2 CSA, and LBM; **Figure 1**). Despite large between-group differences in each outcome there were no meaningful differences in any circulating pre- or post-exercise hormone measured either pre- or post-intervention. Considering steroid hormones are lipid-soluble (e.g., they diffuse across the sarcolemma according to their concentration gradient) it is not surprising that intramuscular T and DHT measured pre- and post-intervention were also not different between HIR and LOR (**Figure 3**). The lack of difference in circulating and intramuscular hormones between HIR and LOR provides evidence that neither hormone delivery to the muscle nor the transfer of steroid hormones inside the muscle are rate-limiting steps in healthy, young individuals.

Androgen receptor content was significantly higher both pre- and post-intervention in the HIR compared to the LOR (**Figure 3**) and was correlated with changes in muscle mass (**Figure 4**). Though another group has found no difference in androgen receptor content between HIR and LOR to RET (Mobley et al., 2018), it is important to acknowledge the differences in study design (e.g., untrained vs. trained participants) and outcome measurements (i.e., cluster analysis based on muscle thickness vs. an aggregate score of DXA and fiber CSA) between them and our work, respectively. The function of an androgen receptor is, when bound with an androgen, to translocate to the nucleus and modify expression of target genes [reviewed elsewhere (Beato and Klug, 2000)], many of which are known targets involved in skeletal muscle growth and development (Wyce et al., 2010). Indeed, when androgen receptors are knocked out in male mice there is a significant reduction in muscle mass and strength (MacLean et al., 2008). Importantly, most steroid hormones have a high affinity with their steroid receptors. For example, the dissociation constant of the androgen receptor to T and DHT is only ∼0.2 to 0.5 nM (Wilson and French, 1976). In the present study, at rest, the molarity of serum T (HIR: 28 ± 7; LOR: 31 ± 7 nM), serum fT (HIR: 0.5 ± 0.01; LOR: 0.5 ± 0.01 nM) and serum DHT (HIR and LOR: 0.7 ± 0.2 nM) all exceeded 0.2–0.5 nM. Given there was no difference in circulating or intramuscular hormones between HIR and LOR, along with high androgen-androgen receptor binding affinity, it seems likely that both at rest and post-exercise existing androgen receptors would have been saturated in skeletal muscle. We hypothesize that though androgen delivery may be a rate-limiting step for RET-induced muscle hypertrophy in hypogonadal men (Bhasin et al., 1997; Kvorning et al., 2013), androgen receptor content is the more important variable in RET-induced androgen-mediated skeletal muscle protein accretion in healthy men (Diver et al., 2003).

### Limitations

We performed 120 correlations in a previous study (Morton et al., 2016) and 48 stepwise regressions here (24 on original data and 24 on the principal components). Applying multiple analyses on the same data was intentional data mining to demonstrate the lack of ability of resting or post-exercise circulating and intramuscular hormones to predict baseline or RET-induced changes in skeletal muscle mass. We could have performed additional statistics to account for multiple testing but this would be uninformative because none of our models explained much variance (as assessed by *R*^2^ values, which did not exceed 0.25). We also acknowledge that although we included a large sample size (*n* = 49) for our systemic hormone analysis we limited ourselves to a relatively smaller sample size (*n* = 20) for our HIR and LOR comparison. We fully admit that in the case of the androgen receptor correlation what we present is an inflated estimate due to the choice of measuring only higher and lower responders to our training protocol. We did our analysis this way to illustrate the difference in RET-induced muscle hypertrophy and investigate the influence of circulating and intramuscular hormone-variables on two distinct groups. Though we were limited by the amount of tissue collected, it is a fair critique that our correlational analysis would be more telling if we included all participants and if we performed additional analyses [e.g., nuclear and cytoplasmic fractions of androgen receptor content as well as multiple gene expressions (Cheung et al., 2017)]. Hence, there is an opportunity for future work to focus on the specific biology that governs androgen receptor regulation and function. Others have postulated that mass spectrometry analysis (as opposed to immunoassays) is necessary to detect small, intramuscular concentrations of steroid hormones (Handelsman and Wartofsky, 2013); however, our intent was to analyze our samples using methods similar to those that others have used in exercise science, which may be dissimilar to those in clinical endocrinology. We recognize that using DXA to measure changes in LBM is not the gold standard, which is why we elected to also include change in type 1 and type 2 fiber CSA to determine our HIR and LOR (Buckinx et al., 2018). In regards to our interpretation, it is naïve to suggest that androgen signaling is exclusively operational via its tendency to bind to an androgen receptor [reviewed elsewhere (Herbst and Bhasin, 2004; Dubois et al., 2012)]. Though transcriptional regulation (e.g., androgen–androgen receptor signaling) is evidenced here as a potent modulator of RET-induced changes in muscle mass, it is also clear that post-transcriptional regulation is at least equally as important for protein synthesis (Schwanhausser et al., 2011) as has been highlighted by recent findings (Figueiredo et al., 2015; Robinson et al., 2017; Mobley et al., 2018) and reviews (Chaillou et al., 2014; McGlory et al., 2017). Lastly, though there is genetic influence that underpins RET-induced skeletal muscle hypertrophy, there are still many environmental considerations, for example consuming adequate dietary protein (Morton et al., 2017), that modulate RET-induced muscle hypertrophy.

## Conclusion

We performed backward elimination and principal component regression on a relatively large cohort (*n* = 49) of resistance-trained men and conclude that the post-exercise AUC (i.e., acute transient net hormonal exposure) and resting hormone concentrations measured in the blood do not share common variance with RET-induced changes in muscle mass. That is, systemic hormone concentrations are not related to, or in any way predictive of, RET-induced changes in muscle mass. Performing subset analysis on the highest- and lowest-responders revealed that androgen receptor content, not intramuscular androgen levels, does not change with RET in trained participants but is significantly higher in HIR than LOR to RET. This study, in conjunction with others (Bamman et al., 2007; Petrella et al., 2008; Davidsen et al., 2011; Eynon et al., 2013), provides evidence that the relative increase in skeletal muscle mass following RET is underpinned by local intramuscular factors and not systemic hormonal concentrations.

## Author Contributions

RM, SO, and SP conceived the research design and conducted the study. RM and MG performed the statistical analyses. PM and SP provided statistical advice. RM, KS, SF, and SP performed data analysis. RM and SP drafted the manuscript. RM, KS, MG, SO, PM, SF, and SP revised and approved the final draft of the manuscript.

## Conflict of Interest Statement

The authors declare that the research was conducted in the absence of any commercial or financial relationships that could be construed as a potential conflict of interest.

## Footnotes

1 http://www.pre.ethics.gc.ca/pdf/eng/tcps2/TCPS_2_FINAL_Web.pdf

## Abbreviations

AUC: area under the curve
CSA: cross sectional area
DHEA: dehydroepiandrosterone
DHT: dihydrotestosterone
fIGF-1: free insulin-like growth factor 1
fT: free testosterone
GH: growth hormone
HIR: high responders
HR: high repetition
IGF-1: insulin-like growth factor 1
LBM: lean body mass
LH: luteinizing hormone
LOR: low responders
LR: low repetition
RET: resistance exercise training
T: testosterone
