# Supplementary material for: Muscle Androgen Receptor Content but Not Systemic Hormones Is Associated With Resistance Training-Induced Skeletal Muscle Hypertrophy in Healthy, Young Men
Source: Front Physiol. 2018 Oct 9;9:1373. doi: 10.3389/fphys.2018.01373 (PMC6189473; doi:10.3389/fphys.2018.01373)
Supplement: Supplementary file 1 [file Table_1.PDF]

Supplementary Material

Muscle Androgen Receptor Content but Not Systemic Hormones is Associated with Resistance Training-Induced Skeletal Muscle Hypertrophy in Healthy, Young Men

Robert W. Morton[1], Koji Sato[2], Michael P. B. Gallaugher[3], Sara Y. Oikawa[1], Paul D. McNicholas[3], Satoshi Fujita[4] and Stuart M. Phillips[1]*

[1]Department of Kinesiology, McMaster University, Hamilton, Canada
[2]Graduate School of Human Development and Environment, Kobe University, Kobe, Japan
[3]Department of Mathematics and Statistics, McMaster University, Hamilton, Canada
[4]Faculty of Sport and Health Science, Ritsumeikan University, Shiga, Japan

*Correspondence:
Prof. Stuart M. Phillips
Department of Kinesiology, McMaster University
1280 Main Street West
Hamilton, ON, CANADA   L8S 4K1
P: 905 525-9140 ext. 24465
E: phillis@mcmaster.ca

Supplementary Table 1. Backwards elimination regression between resting systemic hormones and type 1 CSA, type 2 CSA, and LBM both pre- and post-training.

	Pre-intervention resting					Post-intervention resting			
	Estimate	SEM	t-value	p-value		Estimate	SEM	t-value	p-value
**Pre type 1 CSA**					**Post type 1 CSA**				
Intercept	5449	148	37	<0.001	Intercept	6116	146	42	<0.001
GH	-291	150	-2	0.06	GH	-286	148	-1.9	0.06
	F = 3.80	*df = 47*	*R2 = 0.08*	*pv = 0.06*		*F = 3.76*	*df = 47*	*R2 = 0.07*	*pv = 0.06*
**Pre type 2 CSA**					**Post type 2 CSA**				
Intercept	6194	170	36	<0.001	Intercept	7171	152	47	<0.001
GH	-364	172	-2.1	0.04	fT	-338	154	-2.2	0.03
	F = 4.47	*df = 47*	*R2 = 0.09*	*pv = 0.04*		*F = 4.82*	*df = 47*	*R2 = 0.09*	*pv = 0.03*
**Pre LBM**					**Post LBM**				
Intercept	65	1	62	<0.001	Intercept	66	1	66	<0.001
GH	-1.7	1	-1.6	0.11	T	1.6	1	1.6	0.12
IGF-1	-1.6	1	-1.5	0.14	Lactate	1.7	1	1.7	1.10
	F = 2.30	*df = 46*	*R2 = 0.09*	*pv = 0.11*	Cortisol	1.8	1	1.8	0.09
					IGF-1	1.6	1	1.5	0.14
						F = 2.73	*df = 44*	$R^2 = 0.20$	*pv = 0.04*
