# Supplementary material for: Muscle Androgen Receptor Content but Not Systemic Hormones Is Associated With Resistance Training-Induced Skeletal Muscle Hypertrophy in Healthy, Young Men
Source: Front Physiol. 2018 Oct 9;9:1373. doi: 10.3389/fphys.2018.01373 (PMC6189473; doi:10.3389/fphys.2018.01373)
Supplement: Supplementary file 2 [file Table_2.PDF]

Supplementary Table 2. Principal component regression between post-exercise systemic hormone AUC and the change in each type 1 CSA, type 2 CSA, and LBM.

Pre-intervention post-exercise AUC					Post-intervention post-exercise AUC				
	Estimate	SEM	t-value	p-value		Estimate	SEM	t-value	p-value
**△ Type 1 CSA**					**△ Type 1 CSA**				
Intercept	636	160	4	<0.01	Intercept	669	145	4.6	<0.01
PC7	-259	182	-1.4	0.16	PC5	352	149	2.4	0.02
	F = 2.02	*df = 42*	$R^2 = 0.05$	*pv = 0.16*		*F = 5.63*	*df = 46*	$R^2 = 0.11$	*pv = 0.02*
**△ Type 2 CSA**					**△ Type 2 CSA**				
Intercept	949	178	5.3	<0.01	Intercept	982	181	5.4	<0.01
PC6	-370	192	-1.9	0.06	PC5	521	185	2.8	<0.01
PC7	-638	203	-3.2	<0.01		*F = 7.94*	*df = 46*	$R^2 = 0.15$	*pv < 0.01*
	F = 6.8	*df = 41*	$R^2 = 0.25$	*pv < 0.01*					
**△ LBM**					**△ LBM**				
Intercept	1.2	0.20	6	<0.01	Intercept	1.2	0.20	6.10	<0.01
PC5	0.3	0.20	1.5	0.14					
	F = 2.28	*df = 42*	$R^2 = 0.05$	*pv = 0.14*					
