# Supplementary material for: Muscle Androgen Receptor Content but Not Systemic Hormones Is Associated With Resistance Training-Induced Skeletal Muscle Hypertrophy in Healthy, Young Men
Source: Front Physiol. 2018 Oct 9;9:1373. doi: 10.3389/fphys.2018.01373 (PMC6189473; doi:10.3389/fphys.2018.01373)
Supplement: Supplementary file 3 [file Table_3.PDF]

Supplementary Table 3. Principal component regression between resting hormones and the change in each type 1 CSA, type 2 CSA, and LBM.

Pre-intervention resting					Post-intervention resting				
	Estimate	SEM	t-value	p-value		Estimate	SEM	t-value	p-value
**△ Type 1 CSA**					**△ Type 1 CSA**				
Intercept	667	149	4.5	<0.01	Intercept	667	141	4.7	<0.01
					PC1	-184	106	-1.7	0.09
					PC4	-262	131	-2.0	0.05
						F = 3.52	*df = 46*	$R^2 = 0.13$	*pv = 0.04*
**△ Type 2 CSA**					**△ Type 2 CSA**				
Intercept	978	184	5.3	<0.01	Intercept	978	181	5.4	<0.01
PC7	415	213	2	0.06	PC1	-318	135	-2.4	0.02
	F = 3.81	*df = 47*	$R^2 = 0.08$	*pv = 0.06*		*F = 5.50*	*df = 47*	$R^2 = 0.11$	*pv = 0.02*
**△ LBM**					**△ LBM**				
Intercept	1.2	0.2	6.6	<0.01	Intercept	1.2	0.2	6.7	<0.01
PC3	0.3	0.2	2	0.06	PC1	-0.2	0.1	-1.7	0.11
PC5	-0.4	0.2	-2	0.05	PC2	0.4	0.1	2.6	0.01
	F = 3.89	*df = 46*	$R^2 = 0.15$	*pv = 0.03*		*F = 4.83*	*df = 46*	$R^2 = 0.17$	*pv = 0.01*
