# Supplementary material for: Muscle Androgen Receptor Content but Not Systemic Hormones Is Associated With Resistance Training-Induced Skeletal Muscle Hypertrophy in Healthy, Young Men
Source: Front Physiol. 2018 Oct 9;9:1373. doi: 10.3389/fphys.2018.01373 (PMC6189473; doi:10.3389/fphys.2018.01373)
Supplement: Supplementary file 4 [file Table_4.PDF]

Supplementary Table 4. Principal component regression final output between resting hormones and type 1 CSA, type 2 CSA, and LBM both pre- and post-training.

	Pre-intervention resting					Post-intervention resting			
	Estimate	SEM	t-value	p-value		Estimate	SEM	t-value	p-value
**Baseline type 1 CSA**					**Post type 1 CSA**				
Intercept	5449	159	34	<0.01	Intercept	6116	148	41	<0.01
					PC6	-264	170	-1.6	0.13
						F = 2.43	*df = 47*	$R^2 = 0.05$	*pv = 0.13*
**Baseline type 2 CSA**					**Post type 2 CSA**				
Intercept	6194	174	36	<0.01	Intercept	7171	156	46	<0.01
PC7	-408	197	-2.1	0.04	PC2	-183	123	-1.5	0.14
	F = 4.28	*df = 47*	$R^2 = 0.08$	*pv = 0.04*		*F = 2.21*	*df = 47*	$R^2 = 0.05$	*pv = 0.14*
**Baseline LBM**					**Post LBM**				
Intercept	65	1	63	<0.01	Intercept	66	1	67	<0.01
PC2	1.6	0.8	1.9	0.06	PC5	-3.1	1	-3.1	<0.01
PC3	1.6	0.9	1.7	0.10		*F = 9.47*	*df = 47*	$R^2 = 0.17$	*pv < 0.01*
	F = 3.32	*df = 46*	$R^2 = 0.13$	*pv = 0.05*					
