# Supplementary material for: Muscle Androgen Receptor Content but Not Systemic Hormones Is Associated With Resistance Training-Induced Skeletal Muscle Hypertrophy in Healthy, Young Men
Source: Front Physiol. 2018 Oct 9;9:1373. doi: 10.3389/fphys.2018.01373 (PMC6189473; doi:10.3389/fphys.2018.01373)
Supplement: Supplementary file 5 [file Table_5.PDF]

Supplementary Table 5. Correlations between intramuscular measurements and the change in type 1 CSA, type 2 CSA, and LBM.

	**Δ Type 1 CSA**	**Δ Type 2 CSA**	**Δ LBM**
Pre fT	-0.16	-0.04	-0.33
Post fT	-0.15	0.02	-0.36
Δ fT	-0.03	0.05	0.10
Pre DHT	-0.23	-0.20	-0.23
Post DHT	0.13	0.21	-0.22
Δ DHT	0.27	0.30	0.05
Pre 5a-reductase	-0.33	-0.43	0.24
Post 5a-reductase	-0.04	-0.06	0.43
Δ 5a-reductase	0.24	0.29	0.30
Pre androgen receptor	0.51*	0.61*	0.76*
Post androgen receptor	0.49*	0.65*	0.75*
Δ androgen receptor	0.20	0.39	0.04

*Significantly correlated (P<0.05)
